# Comparing the Nutritional Impact of Dietary Strategies to Reduce Discretionary Choice Intake in the Australian Adult Population: A Simulation Modelling Study

**DOI:** 10.3390/nu9050442

**Published:** 2017-05-03

**Authors:** Jessica A. Grieger, Brittany J. Johnson, Thomas P. Wycherley, Rebecca K. Golley

**Affiliations:** 1 School of Pharmacy and Medical Sciences, University of South Australia, Adelaide, SA 5000, Australia; jessica.grieger@adelaide.edu.au (J.A.G.); brittany.johnson@unisa.edu.au (B.J.J.); 2 School of Health Sciences, Centre for Population Health Research, University of South Australia, Adelaide, SA 5000, Australia; tom.wycherley@unisa.edu.au; 3 Sansom Institute for Health Research, Centre for Population Health Research, Alliance for Research in Exercise, Nutrition and Activity, Adelaide, SA 5000, Australia

**Keywords:** nutrition epidemiology, public health, computer modelling, dietary simulation, discretionary choices, dietary modification, dietary strategies, obesity prevention, chronic disease prevention, adults

## Abstract

Dietary strategies to reduce discretionary choice intake are commonly utilized in practice, but evidence on their relative efficacy is lacking. The aim was to compare the potential impact on nutritional intake of three strategies to reducing discretionary choices intake in the Australian adult (19–90 years) population. Dietary simulation modelling using data from the National Nutrition and Physical Activity Survey 2011–2012 was conducted (*n* = 9341; one 24 h dietary recall). Strategies modelled were: moderation (reduce discretionary choices by 50%, with 0%, 25% or 75% energy compensation); substitution (replace 50% of discretionary choices with core choices); reformulation (replace 50% SFA with unsaturated fats, reduce added sugars by 25%, and reduce sodium by 20%). Compared to the base case (observed) intake, modelled intakes in the moderation scenario showed: −17.3% lower energy (sensitivity analyses, 25% energy compensation −14.2%; 75% energy compensation −8.0%), −20.9% lower SFA (−17.4%; −10.5%), −43.3% lower added sugars (−41.1%; −36.7%) and 17.7% lower sodium (−14.3%; −7.5%). Substitution with a range of core items, or with fruits, vegetables and core beverages only, resulted in similar changes in energy intake (−13.5% and −15.4%), SFA (−17.7% and −20.1%), added sugars (−42.6% and −43%) and sodium (−13.7% and −16.5%), respectively. Reformulating discretionary choices had minimal impact on reducing energy intake but reduced SFA (−10.3% to −30.9%), added sugars (−9.3% to −52.9%), and alcohol (−25.0% to −49.9%) and sodium (−3.3% to −13.2%). The substitution and reformulation scenarios minimized negative changes in fiber, protein and micronutrient intakes. While each strategy has strengths and limitations, substitution of discretionary choices with core foods and beverages may optimize the nutritional impact.

## 1. Introduction

Effective public health nutrition interventions are needed in response to the high prevalence of obesity and chronic diseases globally, and to address the gamut of health, social and economic costs [1,2]. Dietary Guidelines internationally emphasise the selection of nutrient-dense foods whilst limiting ‘discretionary choices’, i.e., foods and beverages that are higher in saturated fat, added sugars, salt and/or empty (nutrient-poor) kilojoules [1,3,4]. In the Australian diet, discretionary choices consistently contribute over one third of daily energy intake [5,6,7], with 98.3% of Australian children and adults consuming on average 34.6% of energy intake from discretionary choices in the most recent national survey [8]. Globally, national survey data shows similar trends, with 89% of US adults consuming discretionary foods [9]; discretionary foods (i.e., non-basic foods high in saturated fats and/or added sugars) contributing 26% to population total energy intake in Mexico [10]; while foods such as hot chips, processed meat, confectionery and soft drinks [11] and sweets and cookies [12] are the highest contributors to energy intake in the UK and Brazil, respectively. Further, diets higher in discretionary choices have been highlighted as less environmentally sustainable [13,14] and in some [15] but not all [16,17] studies were found to be less affordable than healthier diets. Reducing population intakes of discretionary choices is likely to benefit diet quality, health, environmental sustainability and social equity agendas [1].

While policy-based interventions often target discretionary choices [18,19,20], the evidence base on the efficacy of the dietary strategies or nutrition messages underpinning these initiatives is lacking [21,22]. The need for rigorous evaluation of public health nutrition interventions notwithstanding, equally important is the need to use cost-effective and empirical evidence to guide the design of these initiatives or programs [23]. Dietary modelling provides a way to understand and predict behaviours in complex systems [22]. It can bridge the gap between research and policy, either prior to intervention testing or in situations where intervention studies are not feasible [24,25]. Simulation modelling is one form of dietary modelling in which a range of dietary strategies or scenarios can be tested or compared, forecasting via mathematical equations the hypothetical changes in dietary intakes [22]. Modelled estimates, using dietary simulation models, can provide important information to guide policy-based decisions on effective health resource utilisation, for example what dietary strategies or nutrition messages may be effective to take forward in development or testing of public health campaigns.

This paper examines the relative impact of three dietary approaches to reducing population discretionary choices intake: moderation, substitution and reformulation. Moderation targets a reduction in frequency of consumption or reduction in the package/portion size of discretionary choices which will have a direct impact on intakes of energy, saturated fat, added sugars and sodium. However, issues such as the impact on broader nutrient profile, and unintended consequences such as energy compensation have not often been considered in previous simulation studies [22,26]. Substituting or replacing discretionary choices with healthier alternatives may be a more feasible strategy that is likely to have a similar impact on saturated fat, added sugars and sodium intakes, however, the impact on overall nutrient profile has not generally been assessed [22,26]. Moreover, the net impact on energy intake when discretionary choices are replaced with alternative food and beverages has not been examined. Finally, reformulating selected components within food and drinks is appealing because it requires the least change in dietary behaviour. Reformulation of nutrients such as sodium (e.g., within canned foods), added sugars (e.g., within sugar-sweetened beverages) and fat (e.g., within dairy foods) has been widely studied [22,27,28,29,30], typically estimating small reductions at a population level and, at least for sodium, is achievable over the longer term. However, evaluating the impact of reformulation of multiple nutrients while considering feasibility in terms of food technology and consumer taste has rarely been done [21,22].

The current study builds on the gaps identified in the existing literature and models each dietary strategy (moderation, substitution, and reformulation) using the same dataset, with careful consideration of the scenario parameters, as well as considering key assumptions including energy compensation and the method of matching food and drink replacements. The aim of this study was to investigate and compare the relative potential impact of three approaches to reducing discretionary choices on the nutritional intake of the Australian adult population. This research is important to guide policy decisions regarding which set of nutritional messages should underpin policy levers and interventions to address poor dietary patterns in Western countries such as Australia.

## 2. Materials and Methods

Data Source: Base case (i.e., observed) food and nutrient intake data came from the National Nutrition and Physical Activity Survey (NNPAS) 2011–2012 within the Basic Confidentialised Unit Record File microdata [5]. The NNPAS 2011–2012 was conducted between May 2011 and June 2012 in a randomly selected national sample of community dwelling adults, 19–90 years of age, as part of the Australian Health Survey [31]. This study was a secondary analysis using de-identified data, and ethics approval was not required. Food intake (*n* = 9341) was estimated via a single face-to-face 24 h dietary recall administered by trained and experienced interviewers using the Automated Multiple-Pass Method [32]. Friday and Saturday intakes were under-represented due to the low number of recalls performed on Saturdays and Sundays. A nutrient composition database developed specifically for the survey was used to derive nutrient intake and food grouping [33]. Individual dietary intakes were population-weighted using age, gender and geographic location population weights provided by the Australian Bureau of Statistics and aggregated at the individual food code (8-digit) level of the food composition database [33]. The Australian Dietary Guidelines recommend food and beverage choices from the Five Food Groups and to limit discretionary choices. The Five Food Groups (or ‘core choices’) include vegetables, fruits, grain foods, dairy foods and meat and alternatives [1]. Discretionary choices may be higher in saturated fat, added salt, added sugars and/or empty (i.e., nutrient-poor) kilojoules. Examples include cakes, biscuits and pastries, butter, sugary drinks, commercial takeaway foods, confectionary and alcoholic beverages. Discretionary choices were identified using The Australian Bureau of Statistics discretionary choice flag code [34] with all other food and beverages classified as ‘core choices’.

Data Modelling: Simulations were based on a static, microsimulation, discrete time deterministic model. An active data sheet of the observed data (i.e., base case model) from the NNPAS 2011–2012 survey was created in Microsoft Excel with Solver add-in (2013, Microsoft Corporation, Redmond, WA, USA). In brief, aggregated current food and beverage intakes in grams for each individual 8-digit food code from the NNPAS 2011–2012 dataset were combined with per 100 g nutrient values from the survey-specific food composition database to obtain total population nutrient intakes (mean intake/day) [33]. Energy (including dietary fiber) was calculated using the following macronutrient energy densities: Protein 16.736 kJ/g, Carbohydrate 16.736 kJ/g, Fat 37.656 kJ/g, Fiber 8.368 kJ/g, and Alcohol 29.288 kJ/g. To operationalise each simulation modelling scenario, modifiable cells were created to manipulate the observed food and beverage weight (grams), added sugars, sodium and alcohol, or to replace saturated fat with current ratios of unsaturated fats. Discretionary and core choices could be manipulated for foods and beverages separately or combined, and at the collective, food group, or individual (8-digit) food code level. Several validity and quality assurance checks were carried out throughout the development. Internal validity by comparing the output of the base case data sheet against the average per capita nutrient intake profile reported in the NNPAS 2011–2012 survey [5], performance of the modifiable cells with 100% manipulation to ensure the output nutrient profile was adjusting correctly to demonstrate accurate formulas had been inputted, and examination of output results by discretionary choices, core choices and total intake to ensure scenarios had been inputted correctly. Sub-analyses were performed using manipulation at the sub-major food group (3-digit code) or individual 8-digit food code level. The Solver add-in ‘What if’ function (specifically the scenario manager and goal seek commands) was used to run the simulation modelling scenarios to obtain modelled estimates of nutritional data. Primary outcomes were the percent change (from base case intake) in energy intake (including dietary fiber), saturated fat (SFA), added sugars, sodium and alcohol. Secondary outcomes included broader nutritional profiles to capture macronutrients, vitamins and minerals (see list of nutrients reported in Table 1). Nutrient density was expressed as the ratio of nutrients to a standardised unit of energy (i.e., units of nutrients per 1000 kJ). Sensitivity analyses were performed using the same approach to test for parameter uncertainties and assess the external validity of modelling outputs.

*Discretionary choices simulation scenarios:* Literature on previous dietary simulation models [22], discrete dietary intervention strategies [21], and current food science and reformulation [30,35,36] were reviewed to define dietary strategy scenarios targeting a reduction in discretionary choices intake, and to identify key assumptions and parameters for modelling inputs. Details of the moderation, substitution and reformulation scenarios and sensitivity analyses are described below. Because the gram weights of the targeted foods and beverages and their respective replacements were often not equivalent, the replacement items (in grams) were adjusted using a replacement ratio based on population median intake. For example, the median intake ratio of overall discretionary choices relative to core choices was 1.29, this translates to a replacement of 100 g discretionary choices with 129 g core foods. Table S1 provides details of the replacement ratios used to matching variables used in the substitution scenario and the energy compensation sensitivity analyses used in the moderation scenario.

Moderation: The moderation scenario was based on modelling a 50% reduction in energy from all discretionary choices with no food or beverage replacement. A 50% reduction in energy from all discretionary choices would bring the population mean intake close to the top of national nutrition guidelines of 0–1800 kJ (~15% of total energy intake) per day [37]. To account for possible energy compensation [38,39], sensitivity analyses tested a lower and upper bound energy compensation with interquartile ranges of 25% and 75% replacement of energy with all core and discretionary foods. Energy compensation was based on observed base case intake distribution of core and discretionary foods to total energy intake (see Table S1). Energy compensation is less likely to occur following a reduction in liquids [38,40]; therefore, it was assumed a reduction in discretionary *beverages* would result in a direct reduction with no compensation.

Substitution: The first substitution scenario swapped 50% of all discretionary foods for all core foods (including milk), and 50% of all discretionary beverages were swapped for fruit and vegetable juices and water, based on the replacement ratio. Table S1 provides the discretionary choice subgroups and replacement core subgroups, as well as the replacement ratio calculations [41]. For the purpose of this analysis in adults, milk coded as ‘core’ according to the Australian Bureau of Statistics (which includes flavoured and plain milk), was considered suitable as a food substitution rather than as a beverage substitution due to its nutrient profile (calcium, protein), satiating properties, and consumption patterns where/when it is consumed (e.g., on cereal). While flavoured milk is recognised to often be high in added sugars, it was identified as core food according to the Australian Bureau of Statistics, and frequency of consumption in this population was low (4.6%). Alcoholic beverages were replaced with core fruit and vegetable juices or water, using the replacement ratio, as lower alcohol wines or low carbohydrate beers were flagged as discretionary in the database. In the second substitution scenario, 50% of discretionary foods were substituted with fresh/frozen/canned fruit and vegetables (excluding dried fruit, potatoes and legumes), and 50% of discretionary beverages were swapped for fruit and vegetable juices and water, again based on the replacement ratio, per subgroup. This second substitution scenario aimed to choose the least energy-dense options of fruits and vegetables, and is consistent with the Australian Dietary Guidelines which recommends to limit dried fruit [1]. Although the second substitution scenario is less realistic, for example swapping a chocolate bar for an apple, it is still considered feasible and would bring population intakes closer to meeting fruit and vegetable recommendations as well as limiting serves of discretionary choices. In both scenarios, it was assumed that no further energy compensation would result as replacement ratios best reflect current eating patterns.

Reformulation: The reformulation scenario focused on a multi-nutrient reformulation of all discretionary choices by replacing 50% of saturated fat with equivalent volumes of unsaturated fat, and reducing added sugars by 25% and sodium by 20%, as well as a reduction in alcohol content by 25% in alcoholic beverages. Nutrient reformulation targets were based on current industry targets and market examples for subgroups of discretionary choices, taking into account food technology possibilities and implications [30,35,36,42]. The primary targets were within the range of reductions identified: 30% to 75% replacement of saturated fat with unsaturated fat, 10–40% reduction in added sugars and sodium. For example, Pepsico have indicated they will reduce the average amount of saturated fat and added sugars per serving, in certain brands by a respective 15% and 25% by 2020 [30]. Initiatives based on the European Market have indicated a reduction in added sugars in SSB by 10–40% [36], while the previous Australian Health Dialogue indicated to reduce saturated fat by 10% in processed meats and reduce sodium by 10% in various ready to eat cereals and savoury pies [42].

Scenario four incorporated conservative nutrient targets within the constraints of food technology. It was assumed that reformulating fat, sugar and sodium together would maintain palatability and acceptability of products, particularly when levels of target nutrients are slowly reduced and product labelling does not alert consumers of the change. An assumption was also made that portions of the added sugars could be replaced with non-nutritive artificial sweeteners, especially in predominantly sugar-based items such as sweet condiments and sugar-sweetened beverages. Sensitivity analyses were performed to consider the upper and lower bounds of reformulation targets to provide opportunistic and initial targets (highly conservative), respectively. The lower bound targets were replacing 25% SFA with equivalent gram of unsaturated fat, reducing added sugars by 10%, reducing sodium by 10% and alcohol content by 25%, while the upper bounds were replacing 75% saturated fat with equivalent gram of unsaturated fat, reducing added sugars by 40–100%, reducing sodium by 40% and alcohol content by 50%.

## 3. Results

Base case: Population mean daily intakes of energy and nutrients are reported in Table 1. Thirty-five percent of total energy intake came from discretionary choices (25% from foods, 10% from beverages (6% from alcohol)). The nutrient density (per 1000 kJ) of core choices and discretionary choices is shown in Table S2. Per 1000 kJ, 469.1 g of core choices were consumed, whereas 632.3 g of discretionary beverages or 94.4 g of discretionary foods were consumed. Per 1000 kJ discretionary choices, discretionary beverages were higher in total and added sugars, and alcohol, whereas discretionary foods had slightly higher densities for SFA and sodium. Compared to core choices, protein, fiber and micronutrient density was lower in discretionary choices, being 27–64% less nutrient dense per 1000 kJ compared to core choices.

**Figure 1:**
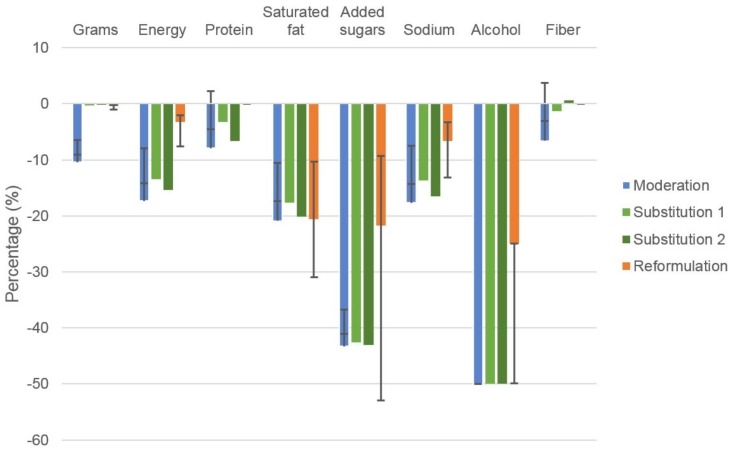
Estimated percentage change in population mean total intake for key nutrient profiles of modelled dietary strategies to reduce discretionary choices. Results are expressed as a percentage change in total energy intake. Error bars represent the sensitivity analyses performed for moderation and reformulation simulations, no sensitivity analyses were performed for substitution scenarios. Moderation: Reduction of discretionary choices by 50% with no energy compensation. Substitution: Replacement of 50% of discretionary choices with 1: core foods, water, fruit/vegetable juices; 2: fruit, vegetables, water, fruit/vegetable juices. Reformulation: replacing 50% of SFA with unsaturated fat, reduce added sugar by 25%, sodium by 20%, and alcohol by 25% in discretionary choices.

Moderation scenario and sensitivity analyses: Figure 1 (blue bars) shows the impact of reducing discretionary choices by 50%, with the sensitivity analyses (25% and 75% energy compensation). The complete dietary profile is reported in Table S3, along with sensitivity analysis shown in Table S4. Moderating discretionary choices resulted in a 10.4% lower total intake quantity (grams) (sensitivity analyses range: 25% energy compensation −9.1%; 75% energy compensation −6.5%) and 17.3% lower total energy intake (−14.2%, −8.0%) compared with the base case. Compared to the base case intake, modelled intakes were lower for SFA by 20.9% (−17.4%, −10.5%), added sugars by 43.3% (−41.1%, −36.7%), sodium by 17.7% (−14.3%, −7.5%), and alcohol by 50.0% (−50.0%, −50.0%). Protein and fiber intake was also lower by a respective 7.9% (−4.5%, +2.3%) and 6.6% (−3.1%, +3.7%). Modelled micronutrient intakes were lower compared to the base case by −6.9% for folate (−3.0%, +2.8%), up to −12.7% (−9.8%, −4.0%) for vitamin B6. All micronutrients were reduced in the 25% energy compensation whereas the 75% energy compensation increased several micronutrients including fiber, vitamin A retinol equivalents, dietary folate equivalents, calcium, iodine, zinc and copper compared to the base case.

**Figure 2:**
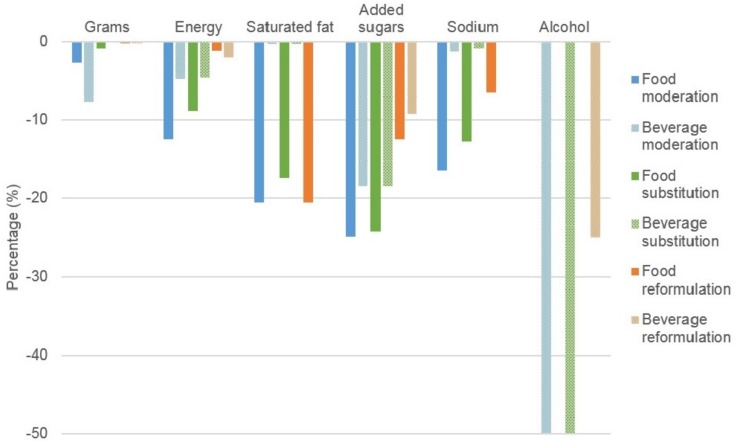
Estimated impact of simulations to moderate, substitute and reformulate on discretionary foods or beverages on mean population intake of target nutrients. Results are expressed as a percentage change in total energy intake. Food moderation: Reduction of discretionary foods by 50% with no energy compensation. Beverage moderation: Reduction of discretionary beverages by 50% with no energy compensation. Food substitution: Replacement of 50% of all discretionary foods with all core foods. Beverage substitution: Replacement of 50% of discretionary beverages with water, fruit/vegetable juices. Food reformulation: Reformulation of all discretionary foods by replacing 50% SFA with equivalent gram of unsaturated fat, reducing added sugars by 25%, reducing sodium by 20%. Beverage reformulation: Reformulation of all discretionary beverages by reducing added sugars by 25%, reducing sodium by 20%, reducing alcohol by 25%.

Figure 2 (blue bars) shows the modelled intake separately for a 50% reduction in discretionary foods compared to a 50% reduction in discretionary beverages. Compared to base case, there was a greater decrease in intake of energy, SFA and sodium following a decrease in intake of discretionary foods compared to discretionary beverages, whereas the decrease in weight of beverages was larger (Table 2). Table S5 demonstrates the impact of incorporating a 25% and 75% energy compensation to include all core and discretionary foods, while simultaneously reducing discretionary foods by 50%. Compared to base case, the modelled intake of grams changed by −1.4% and +1.2%, and reduced energy intake by −9.4% and −3.1%, respectively. The smaller 25% energy compensation maintained the reduced intake of macronutrients and micronutrients (except for vitamin C), while the larger 75% energy compensation generally reduced intake of macronutrients but increased micronutrients (Table S5).

Substitution scenarios and sensitivity analysis: Figure 1 shows replacement of 50% of discretionary foods with core foods and 50% of discretionary beverages with water, fruit and vegetable juices based on replacement ratio (light green bars); and replacement of 50% of discretionary foods with fruit and vegetables and 50% of discretionary beverages with water, fruit and vegetable juices based on replacement ratio (dark green bars). Both scenarios resulted in similar reductions from the base case of −0.3% and −0.2%, and −13.5% and −15.4% for gram and energy intake respectively which was also similar in magnitude to the reduction achieved through moderation (with 0% energy compensation). Between the substitution and moderation scenarios, similar differences in the modelled compared to base case intakes were observed for SFA, added sugars, sodium, and alcohol. The difference in protein was −3.2% when substituting a range of core foods compared to −6.6% when only fruits and vegetables were targeted. The difference in nutrients compared to the base case intake were also less for the substitution scenario incorporating a range of core foods (range +0.8% for vitamin C to −8.4% for vitamin B6) compared to the substitution scenario targeting just core fruit and vegetables (range +7.9% vitamin C to −9.4% for vitamin E).

The estimated impact of substituting discretionary foods or beverages on mean population intake of target nutrients is shown in Table 2. Substituting half of all discretionary foods to all core foods (Figure 2), produced a similar −0.9% change in grams of intake to replacing all discretionary choices (Figure 1). The impact of substituting half of all discretionary beverages with water, or fruit and vegetable juices (Figure 2) demonstrates a similar change in energy and added sugars compared to replacement of all discretionary foods (Figure 2) or all discretionary choices (Figure 1). When only discretionary water-based beverages are replaced with water, or fruit and vegetable juices, the reduction in energy intake and added sugars is smaller again (Table S6), albeit similar to the changes when the same beverages are replaced to water (Table S6).

Reformulation scenarios and sensitivity analyses: Table S3 shows the modelled intake when reformulating discretionary choices by replacing 50% of SFA with unsaturated fat, reducing added sugars and alcohol by 25% and reducing sodium by 20%, and sensitivity analyses are shown in Table S4. Compared to the base case, modelled intakes were 0.4% (−0.2%, −1.0%) and 3.3% (−2.1%, −7.6%) lower in grams and energy intake respectively (Figure 1; orange bars). Modelled intake of SFA was 20.6% (−10.3%, −30.9%) lower than the base case intake, with similar differences observed for added sugars, 21.7% (−9.3%, −52.9%), and a 25.0% (−25.0% to −49.9%) lower modelled intake of alcohol. Modelled sodium intake was 6.6% (−3.3% to −13.2%) lower than the base case scenario. As expected, no difference in the modelled intake of other macro- and micro-nutrient intakes were observed compared to the base case. Likewise, as shown in Table 2, when reformulating either foods or beverages), the reductions in SFA and sodium was most evident when reformulating foods, the reduction in alcohol occurred mostly when reformulating *beverages*, while the reduction in added sugars occurred in both (Figure 2; Table S7 for full nutrient profile and sensitivity analyses).

## 4. Discussion

This study aimed to model three dietary strategies that target discretionary food and beverage intake, and to compare the nutritional impact in the Australian adult population. The moderation and substitution scenarios demonstrated similar theoretical reductions in energy (~15%), SFA (~20%), sodium (~15%), added sugars (~43%) and alcohol (50%) intake. The substitution scenario that targeted replacement with a wide range of core foods mitigated reductions in protein, fiber and micronutrient intakes by maintaining total food intake. While the reformulation scenario had little impact on protein, fiber and micronutrient intakes and produced a similar reduction in SFA (~20%) to the moderation and substitution scenarios, the reduction in total energy intake, added sugars, sodium and alcohol were smaller by around one third to one half compared to the moderation and substitution scenarios. The substitution dietary strategy has the best potential, at least theoretically, to reduce energy intake and less healthful nutrients whilst maintaining or improving micronutrient and fiber intakes.

This study makes a unique contribution to the literature by demonstrating the impact of substitution of discretionary choices with a range of core foods and beverages. Replacement of discretionary choices with only fruits, vegetables and core beverages; or a broad range of core foods (i.e., also including meats, dairy, grains) and core beverages, achieved comparable maintenance of total food consumed and a similar reduction of around 43% in added sugars. The former strategy modelled a slightly greater reduction in SFA and sodium intakes, but nutrients such as calcium, zinc, iodine and B vitamins were reduced to a greater extent than when replacing with a broad range of core choices. There have been few previous studies that have modelled a replacement of a range of unhealthy foods with a choice of healthier alternatives [26,41,43]. Overall, these studies estimated small reductions in SFA and sucrose with complete substitution from higher fat or higher sugar products to lower fat or lower sugar varieties [26]; estimated reductions in energy intake and improved diet quality with 25%, 50%, 75% and 100% substitutions from discretionary choices to healthy food options/varieties [41], and estimated a reduction in deaths from coronary heart disease and stroke with substitution from one unhealthy snack to a healthy snack [43]. It is evident from the current study that replacement with a range of core foods and beverages produces important changes to the nutrient profile, and that fruits and vegetables are key foods that should be included in the replacement.

Previous studies using moderation have generally focussed on reducing sugar-sweetened beverages [26,44,45,46,47], or selected ‘unhealthy’ foods, such as pastries, sweets, crisps, sausages, and fatty meats [26,48,49,50]. The study by Sacks et al. [49] moderated intake of beverages and foods, which led to a decrease in adult population energy intake by ~150 kJ/day compared to ~1500 (range 600–1230) kJ/day in the present study, with moderation of all discretionary choices consumed in the study population, and depending on degree of energy compensation assumed. This difference reflects the discrete study aims, with Sacks et al. [49] modelling the impact of a 10% tax on unhealthy food, compared to the current study’s modelling the impact of reducing discretionary choices intake via a reduction in actual food intake. Our study also demonstrated modelled reductions in intakes of protein and fiber of 6–8%, compared to the base case. This may be the result of reductions in cereal based discretionary foods such as meat pies, quiche, muffins, burgers and pizza. The base case intake of protein as percentage of energy was 17.5%, which is at the lower end of the acceptable macronutrient distribution [51] and mean intakes of fiber (22.9 g) is lower than the adequate intake for males and females [51]. Individual targeting of foods to moderate may be required for those with low intakes of protein or fiber, or, including additional core and discretionary foods at current intake ratios as demonstrated in the 75% energy compensation analysis, may be practical, as this has estimated increases of 2–4% in protein and fiber. Overall, the moderation scenario was effective in reducing total food and energy intake, even allowing for energy compensation. Naturally, reductions in beneficial micronutrients occurred as a result of reduced food intake, however large reductions were estimated for SFA, sodium, added sugars and alcohol. This reduction in alcohol is equivalent to around three quarters of a standard drink, highlighting that alcoholic beverages are also an important target in moderating discretionary choice (and energy) intake in adults.

In order to make such dietary changes, several policy levers will be required to achieve the degree of change in population discretionary choice intake. For example, taxes on SSB have been shown to have reasonable effects in moderating intake of SSB and energy intake [44,45,46,52,53], and reducing body weight [45,46,54], while value added taxes and/or subsidies to promote fruit and vegetable intake estimated marginal intakes in fruit and vegetables [55,56]. Food labelling strategies have been shown to be effective for substitution to healthier food choices, reducing body weight [49,57], energy intake [49,57,58], and reducing SFA, added sugars and sodium [58,59,60]. Policies aimed at mandatory reformulation in Westernized countries were also effective in reducing intakes of sodium, SFA and added sugars, but also a range of health-related outcomes (reviewed in [35]), but importantly, the health and economic benefits of investing in such reformulation programs has been established [35]. The economic benefits were less clear in tax and subsidy studies, however may be more beneficial in low socioeconomic regions [61]. Nevertheless, it is likely that a combination of voluntary and legislated approaches is required to achieve effective dietary changes from these strategies, along with individual behavioural changes to enable synergistic effects.

The present study aimed to address key methodological limitations identified in previous simulation studies, including the impact on complete dietary profile along with consideration of potential for energy compensation [21,22]. Sensitivity analyses were undertaken, incorporating some additional food and beverage allowance in the moderation scenario, on the assumption that energy compensation as a result of moderation of discretionary choice intake was possible and perhaps likely. Modelling that assumed unintended or undirected energy compensation (ranging from 25% to 75% of the reduction in discretionary choice energy intake), estimated reductions in energy, SFA, added sugars and sodium of ~10%, and up to 40% for added sugars and alcohol. These sensitivity analyses provide confidence in moderation as a useful intervention message. However, the substitution strategy also directly addresses the potential for energy compensation with added benefits on overall diet quality via an increase in core choices.

Previous reformulation studies have typically targeted single foods and nutrients [22]. The present study estimated changes in intake of multiple target nutrients with reformulation parameters identified in the literature. It was assumed that reformulating foods in gradual amounts over time may minimise consumer awareness and negative attitudes to “new products”, assuming taste and palatability is preserved, thereby maintaining food purchasing and consumption patterns [62]. The flow-on effect of manipulating one nutrient relative to another was also considered. It was deemed feasible that modest reductions across all nutrients (i.e., SFA, added sugars, sodium) will allow for a balance in sweet and savoury flavours to maintain similar flavour profiles. Not surprisingly, small changes in nutrients were observed in our reformulation scenarios, as it is evident that manipulating nutrient content in foods can only be achieved in gradual amounts and over long periods of time. The current study manipulated a range of nutrients in the same foods, which estimated marginal reductions in energy intake, but clearly improved the nutrient profile of the Australian population, specifically reducing SFA, added sugars, alcohol, and had a small reduction in sodium. Such results not only suggest that discretionary foods could be targeted in relation to sodium content, but also that reducing the sodium content of core choices, particularly breads and cereals, will further lower sodium intake. Although the reformulation strategy produced smaller changes in reducing added sugars and sodium than the moderation and substitution strategies, reformulation is an appropriate and feasible strategy, as modest reductions across all nutrients will likely maintain product palatability and acceptability, thereby less likely influencing any changes in food consumption behaviour.

Strengths of this study include the systematic method for altering dietary intakes that can be used to inform intervention studies. Thorough sensitivity analyses and inclusion of foods beyond the minimum to meet nutrient requirements, the current modelling provides a range of potential outcomes and reveals the logical connection between inputs (i.e., data and assumptions) and outputs. The current study also followed key criteria relevant to dietary modelling regarding model structure, model performance, and transparency, and did not restrict changes in one or a few foods or nutrients, but explored all discretionary choices and the complete nutrient profile. We based the substitution scenarios on a ratio to match the discretionary choices and core replacements, as the purpose was to not maintain isocaloric intake (which would occur with a kJ for kJ swap), and substituting by gram intake could be unfeasible, particularly when substituting high-volume foods (e.g., popcorn). Limitations include the use of one-day dietary intake, thus not reflecting usual intake, along with a low sample of food records collected for Friday and Saturday [63]. This would likely underrepresent days where high intake of discretionary choices might be consumed. Misreporting is also a limitation, but as it is unlikely all items were misreported equally (e.g., fruit and vegetables over reported, discretionary choices under reported), applying a blanket underreporting factor was not deemed appropriate in our modelling scenarios. The current study assumed that the whole population would make such changes and preferences and consumption patterns would not differ; however, the use of sensitivity analyses demonstrating upper and lower bounds highlights changes in nutrients where population changes may be less feasible. Finally, the dietary modelling is a cross-sectional analysis thus extrapolating to changes in longer term intakes is not possible.

The study findings have implications for policy and practice. The modelled discretionary foods and beverages changes would achieve population level limits (i.e., ~2.5 servings per day or ~15% of total energy intake), down from the current 35% of total energy intake. Serving as a benchmark, the study findings are useful for guiding future research, as well as policy decisions. It is unlikely that any single policy lever (e.g., front of pack labelling or a sugary drink tax in isolation) will achieve the necessary change in discretionary choice intake. Future modelling could consider the additive or synergistic effect of a range of policy levers (e.g., nutrient profiling and front of pack labelling, combined with nutrition promotion/education campaigns, or use of taxation measures) that would achieve recommended limits. Research of the downstream effects on overall diet quality, compliance with dietary guidelines and health outcome is also warranted.

## 5. Conclusions

The substitution dietary strategy has the best potential, at least theoretically, to reduce energy intake and less healthful nutrients such as SFA, added sugars and sodium whilst maintaining or improving protein, fiber and micronutrient intakes. Reformulated products may play a role in improving less healthful nutrient intakes where energy moderation is not a priority, but is unlikely to contribute to obesity prevention efforts. Future dietary modelling research could test the nutritional impact of a combination of dietary strategies, and whether the current findings are similar in sub-populations such as obese or disadvantaged groups. The present study can aid decision makers to prioritize and deliver policy interventions. While each strategy has strengths and limitations, substitution of discretionary choices with a range of core food appears to balance maximizing nutritional impact and ease of adoption in populations.

## Figures and Tables

**Table 1:** Australian Health Survey population weighted mean base case intake of adults aged 19 year and over.

	Total Intake ^1^ Original Intake	Core Choices Original Intake	Discretionary Choices ^2^ Original Intake	Discretionary Foods Original Intake	Discretionary Beverages Original Intake ^3^
Grams (g)	3337.7	2652.2	685.5	178.8	513.6
Energy (kJ)	8697.8	5654.0	3043.8	2173.5	841.9
Protein (g) (%E ^4^)	91.0 (17.5)	75.6 (14.6)	15.4 (3.0)	13.5 (2.6)	0.9 (0.2)
Total fat (g) (%E)	73.8 (32.0)	47.4 (20.5)	26.4 (11.4)	26.1 (11.3)	0.3 (0.1)
Saturated fat (g) (%E)	27.7 (12.0)	16.1 (7.0)	11.6 (5.0)	11.4 (4.9)	0.2 (0.1)
Carbohydrate (g) (%E)	225.9 (43.5)	145.6 (28.0)	80.4 (15.5)	56.3 (10.8)	23.6 (4.5)
Total sugars (g)	102.9 (19.8)	51.3 (9.9)	51.5 (9.9)	30.7 (5.9)	20.6 (4.0)
Added sugars (g) (%E)	50.6 (9.7)	6.7 (1.3)	43.9 (8.4)	25.2 (4.9)	18.6 (3.6)
Free sugars (g) (%E)	57.8 (11.1)	10.8 (2.1)	47.1 (9.1)	26.8 (5.2)	20.2 (3.9)
Sodium (mg)	2430.5	1567.1	863.5	796.8	61.6
Alcohol (g) (%E)	14.4 (4.8)	0.0 (0.0)	14.4 (4.8)	0.0 (0.0)	14.4 (4.8)
Fiber (g)	22.9	19.9	3.0	2.9	0.1
Vitamin A retinol equivalents (µg)	851.8	732.2	119.6	107.3	7.9
Thiamine (vitamin B1) (mg)	1.5	1.2	0.3	0.3	0.0
Riboflavin (vitamin B2) (mg)	1.9	1.5	0.4	0.3	0.1
Niacin equivalents (mg)	41.4	33.5	7.9	6.2	1.4
Dietary folate equivalent (µg)	609.9	529.0	80.9	74.1	4.8
Vitamin B6 (pyridoxine) (mg)	1.5	1.1	0.4	0.2	0.2
Vitamin B12 (cobalamin) (µg)	4.5	3.8	0.7	0.6	0.1
Vitamin C (mg)	102.3	86.1	16.2	3.9	12.1
Vitamin E (mg)	10.5	7.8	2.7	2.6	0.1
Calcium (mg)	804.6	677.6	127.0	93.4	25.1
Iodine (µg)	172.3	146.5	25.8	17.9	6.5
Iron (mg)	11.1	9.0	2.2	1.7	0.4
Magnesium (mg)	338.7	274.2	64.5	40.6	21.4
Phosphorous (mg)	1466.9	1137.3	329.6	259.8	58.6
Potassium (mg)	2912.5	2345.5	567.0	413.2	141.4
Selenium (µg)	91.0	75.4	15.6	12.9	2.5
Zinc (mg)	11.0	9.3	1.7	1.5	0.1

^1^ Total intake includes all core and discretionary foods and beverages; ^2^ Discretionary choices includes all discretionary foods and beverages (including meal replacements and sports products); ^3^ Discretionary beverages excludes meal replacements and sports products such as protein shakes and gels; ^4^ Percentage of total energy intake.

**Table 2:** Estimated impact of moderating, substituting and reformulating discretionary foods or beverages on mean population intake of target nutrients.

	Moderation of All Discretionary Foods by 50%	Moderation of All Discretionary Beverages by 50%	Replacement of All Discretionary Foods with All Core Foods ^2^	Replacement of Discretionary Beverages with Water, or Fruit and Vegetable Juices ^3^	Reformulate All Discretionary Foods ^4^	Reformulate All Discretionary Beverages ^5^
Grams (g)	3248.3	3080.9	3306.9	3357.3	3331.2	3329.5
Energy (kJ)	7610.8	8276	7924.3	8293.9	8592.2	8513.9
Saturated fat (g) (% E ^1^)	22.0 (10.9%)	27.6 (12.6%)	22.9 (10.9%)	27.6 (12.5%)	22.0 (9.6%)	27.7 (12.3)
Added sugars (g) (% E)	38.0 (8.4%)	41.3 (8.4%)	38.3 (8.1%)	41.3 (8.3%)	44.3 (8.6%)	46.0 (9.0)
Sodium (mg)	2032.1	2399.7	2118.7	2408.8	2271.2	2429.8
Alcohol (g) (% E)	14.4 (5.5%)	7.2 (2.6%)	14.4 (5.3%)	7.2 (2.6%)	14.4 (4.9%)	10.8 (3.7)

^1^ Modelled nutrient percentage of total energy intake; ^2^ Replacement of 50% of all discretionary foods with all core foods (including milk) based on a replacement ratio; ^3^ Replacement of 50% of discretionary beverages with water, or fruit and vegetable juices based on a replacement ratio; ^4^ Reformulate all discretionary foods by replacing 50% saturated fat with equivalent gram of unsaturated fat, reducing added sugars by 25% and reducing sodium by 20%; ^5^ Reformulate all discretionary beverages by reducing added sugars by 25% and reducing sodium by 20% and reducing alcohol by 25%.

## Supplementary Materials

The following are available online at www.mdpi.com/2072-6643/9/5/442/s1, Table S1: Moderation scenarios energy compensation ratios and substitution scenarios replacement ratios, Table S2: Australian Health Survey population weighted mean base case intake nutrient density (amount of nutrient/1000 kJ) of adults aged 19 year and over, Table S3: Modelled intakes simulating the impact on population mean nutrient profile of dietary strategies to reduce discretionary choices in Australian adults, Table S4: Sensitivity analyses testing the impact of manipulating discretionary choices intake on population mean nutrient profile, Table S5: Sensitivity analyses testing the impact of moderating discretionary foods on population mean nutrient profile, Table S6: Sensitivity analyses testing the impact of substituting water-based discretionary beverages to core beverages on population mean nutrient profile, Table S7: Sensitivity analyses testing the impact of reformulating discretionary foods or beverages on population mean nutrient profile.
