# Correction: Grieger, J.A.; et al.: Comparing the Nutritional Impact of Dietary Strategies to Reduce Discretionary Choice Intake in the Australian Adult Population: A Simulation Modelling Study. *Nutrients* 2017, *9*, 442

**DOI:** 10.3390/nu9080851

**Published:** 2017-08-09

**Authors:** Jessica A. Grieger, Brittany J. Johnson, Thomas P. Wycherley, Rebecca K. Golley

**Affiliations:** 1 School of Pharmacy and Medical Sciences, University of South Australia, Adelaide, SA 5000, Australia; jessica.grieger@adelaide.edu.au (J.A.G.); brittany.johnson@unisa.edu.au (B.J.J.); 2 School of Health Sciences, Centre for Population Health Research, University of South Australia, Adelaide, SA 5000, Australia; tom.wycherley@unisa.edu.au; 3 Sansom Institute for Health Research, Centre for Population Health Research, Alliance for Research in Exercise, Nutrition and Activity, Adelaide, SA 5000, Australia

We would like to submit the following correction to our recently published paper [1] because there was an error in the median intake ratio calculation used in the substitution strategies. The median intake ratios have been corrected throughout Supplementary Table S1. The corrected calculations have led to minor changes (i.e., less than 5% change in nutrient intakes) to results reported in the abstract (page 1), the substitution results text (page 8 and 9), tables and figures (Table 2, columns 4 and 5, page 8; Figure 1, light green and dark green bars, page 6; Figure 2, green bars page 7; Supplementary Table S1, substitution replacement rows; Supplementary Table S3, columns 4–8; and Supplementary Table S6, columns 2 and 3). The corrected tables and text are shown below. These changes have no material impact on the conclusions of our paper. We apologize for any inconvenience caused. The manuscript will be updated and the original will remain online on the article website.

## Abstract

Substitution with a range of core items, or with fruits, vegetables and core beverages only, resulted in similar changes in energy intake (−8.8% and −13.6%), SFA (−13.4% and −19.4%), added sugars (−41.7% and −42.7%) and sodium (−9.0% and −15.6%), respectively.

## Results

Both scenarios resulted in similar changes from the base case of +0.9% and +0.6%, and −8.8% and +3.6% for gram and energy intake respectively.

The difference in protein was −3.0% when substituting a range of core foods compared to −5.4% when only fruits and vegetables were targeted. The difference in nutrients compared to the base case intake were also less for the substitution scenario incorporating a range of core foods (range—3.0% for vitamin B6 to +5.0% for fiber and DFE compared to the substitution scenario targeting just core fruit and vegetables (range—7.9% for vitamin B 12 to +18.4% for vitamin C).

Substituting half of all discretionary foods to all core foods (Figure 2), produced a similar +0.9% change in grams of intake to replacing all discretionary choices (Figure 1).

## Figures and Tables

**Figure 1:**
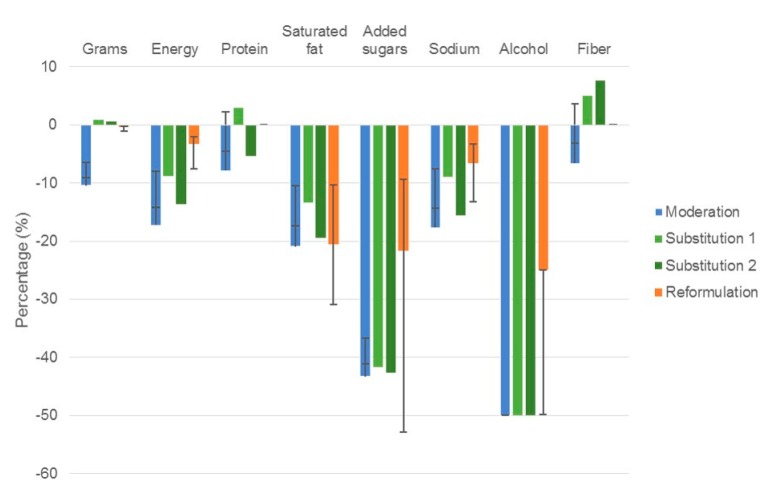
Estimated percentage change in population mean total intake for key nutrient profiles of modelled dietary strategies to reduce discretionary choices. Results are expressed as a percentage change in total energy intake. Error bars represent the sensitivity analyses performed for moderation and reformulation simulations, no sensitivity analyses were performed for substitution scenarios. Moderation: Reduction of discretionary choices by 50% with no energy compensation. Substitution: Replacement of 50% of discretionary choices with 1: core foods, water, fruit/vegetable juices; 2: fruit, vegetables, water, fruit/vegetable juices. Reformulation: replacing 50% of SFA with unsaturated fat, reduce added sugar by 25%, sodium by 20%, and alcohol by 25% in discretionary choices.

**Figure 2:**
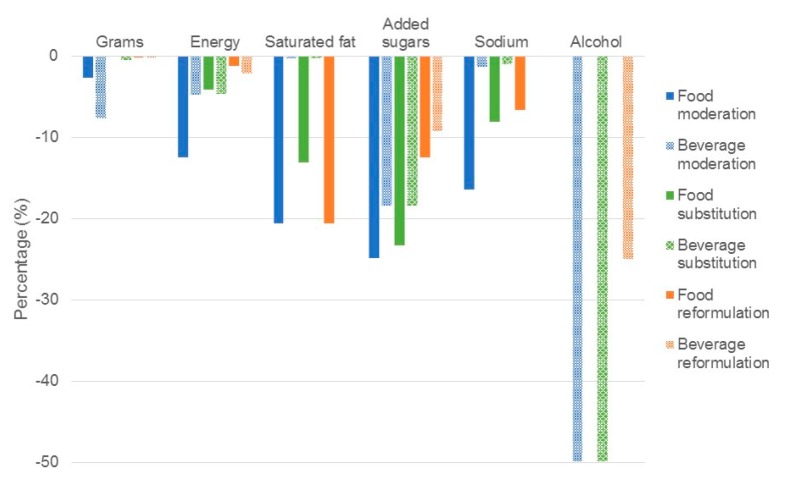
Estimated impact of simulations to moderate, substitute and reformulate on discretionary foods or beverages on mean population intake of target nutrients. Results are expressed as a percentage change in total energy intake. Food moderation: Reduction of discretionary foods by 50% with no energy compensation. Beverage moderation: Reduction of discretionary beverages by 50% with no energy compensation. Food substitution: Replacement of 50% of all discretionary foods with all core foods. Beverage substitution: Replacement of 50% of discretionary beverages with water, fruit/vegetable juices. Food reformulation: Reformulation of all discretionary foods by replacing 50% SFA with equivalent gram of unsaturated fat, reducing added sugars by 25%, reducing sodium by 20%. Beverage reformulation: Reformulation of all discretionary beverages by reducing added sugars by 25%, reducing sodium by 20%, reducing alcohol by 25%.

**Table 2:** Estimated impact of moderating, substituting and reformulating discretionary foods or beverages on mean population intake of target nutrients.

	Moderation of All Discretionary Foods by 50%	Moderation of All Discretionary Beverages by 50%	Replacement of All Discretionary Foods with All Core Foods ^2^	Replacement of Discretionary Beverages with Water, or Fruit and Vegetable Juices ^3^	Reformulate All Discretionary Foods ^4^	Reformulate All Discretionary Beverages ^5^
Grams (g)	3248.3	3080.9	3384.7	3320.8	3331.2	3329.5
Energy (kJ)	7610.8	8276	8341.1	8291.9	8592.2	8513.9
Saturated fat (g) (%E ^1^)	22.0 (10.9%)	27.6 (12.6%)	24.1 (10.9%)	27.6 (12.5%)	22.0 (9.6%)	27.7 (12.3)
Added sugars (g) (%E)	38.0 (8.4%)	41.3 (8.4%)	38.8 (7.8%)	41.3 (8.3%)	44.3 (8.6%)	46.0 (9.0)
Sodium (mg)	2032.1	2399.7	2233.9	2407.3	2271.2	2429.8
Alcohol (g) (%E)	14.4 (5.5%)	7.2 (2.6%)	14.4 (5.0%)	7.2 (2.6%)	14.4 (4.9%)	10.8 (3.7)

^1^ Modelled nutrient percentage of total energy intake. ^2^ Replacement of 50% of all discretionary foods with all core foods (including milk) based on a replacement ratio. ^3^ Replacement of 50% of discretionary beverages with water, or fruit and vegetable juices based on a replacement ratio. ^4^ Reformulate all discretionary foods by replacing 50% saturated fat with equivalent gram of unsaturated fat, reducing added sugars by 25% and reducing sodium by 20%. ^5^ Reformulate all discretionary beverages by reducing added sugars by 25% and reducing sodium by 20% and reducing alcohol by 25%.

## Supplementary Materials

The following are available online at www.mdpi.com/2072-6643/9/8/851/s1, Table S1: Moderation scenarios energy compensation ratios and substitution scenarios replacement ratios, Table S2: Australian Health Survey population weighted mean base case intake nutrient density (amount of nutrient/1000 kJ) of adults aged 19 years and over, Table S3: Modelled intakes simulating the impact on population mean nutrient profile of dietary strategies to reduce discretionary choices in Australian adults, Table S4: Sensitivity analyses testing the impact of manipulating discretionary choices intake on population mean nutrient profile, Table S5: Sensitivity analyses testing the impact of moderating discretionary foods on population mean nutrient profile, Table S6: Sensitivity analyses testing the impact of substituting water-based discretionary beverages to core beverages on population mean nutrient profile, Table S7: Sensitivity analyses testing the impact of reformulating discretionary foods or beverages on population mean nutrient profile.
