# Supplementary material for: Correction: Grieger, J.A.; et al.: Comparing the Nutritional Impact of Dietary Strategies to Reduce Discretionary Choice Intake in the Australian Adult Population: A Simulation Modelling Study. Nutrients 2017, 9, 442
Source: Nutrients. 2017 Aug 9;9(8):851. doi: 10.3390/nu9080851 (PMC5579644; doi:10.3390/nu9080851)
Supplement: Supplementary file 1 [file nutrients-09-00851-s001.zip › nutrients-217719-supplementary.pdf]

SUPPLEMENTARY FILES

Supplementary Table S1: Moderation scenarios energy compensation ratios and substitution scenarios replacement ratios

**Moderation energy compensation**			
**Scenario**	**Discretionary foods contribution**	**Core foods contribution**	**Energy compensation**
Reduce discretionary foods by 50% with 25% energy compensation from all choices based on current intake distribution.	Energy from discretionary foods 2173.5kJ divided by energy from all foods 7593.5kJ = 0.2862 (28.6%)	Energy from core foods 5420.0kJ divided by energy from all foods 7593.5kJ = 0.7137 (71.4%)	25% of energy deficit (1087.02kJ) = 271.76kJ of which 77.72kJ from discretionary foods and 194.03kJ from core foods
Reduce discretionary foods by 50% with 75% energy compensation from all choices based on current intake distribution.	As above	As above	75% of energy deficit (1087.02kJ) = 815.27kJ of which 233.17kJ from discretionary foods and 582.10kJ from core foods

**Substitution replacement**			
**Scenario**	**Discretionary choices to be substituted**[1]	**Replacement core choices**[2]	**Replacement ratio**[3]
Discretionary foods			
Replace 50% of grams of discretionary foods (89.4g) with 127.9g of fresh/frozen/canned fruit and vegetables (excludes dried fruit, potatoes and legumes) matching using replacement ratio of 1.431.	Sweet and savoury buns, high-sugar ready-to-eat breakfast cereals, biscuits (cookies), cakes and muffins, crackers, sweet/savour pastries, commercial fried food and takeaway (pizza, hamburger), butter and other solid fats, sausages, processed meats, cream, ice cream, dry soup mix, sauces, hot fried potato, potato crisps, savoury snacks, sugar, jams and honey, desserts, confectionary, fruit and muesli bars, vegemite, stock, salt	Pome fruit, berry fruit, citrus fruit, stone fruit, tropical and subtropical fruit), other fruit, mixtures of fruit or mixed dishes where fruit is the major component<br><br>Cabbage, cauliflower and similar brassica vegetables, carrot and similar root vegetables, leaf and stalk vegetables, peas and beans, tomato and tomato products, other fruiting vegetables, other vegetables and vegetable combinations, dishes where vegetable is the major component	Population discretionary median intake = 0.0568<br><br>Population core median intake = 0.0813<br><br>1.431

Replace 50% of all discretionary foods (89.4g) with 136.4g of all core foods (i.e. fruit and vegetables (including potatoes, legumes, dried fruit), breads and cereals, dairy (including milk), meat and alternatives, core fats and oils, and core sauces, soups, condiments) matching using replacement ratio of 1.526.	As above	As above with the addition of the following:<br><br>Dried fruit, preserved fruit<br><br>Mature legumes and pulses, mature legume and pulse products and dishes, potatoes<br><br>Milk, yoghurt, cheese and/or their alternatives (soy, rice, oat, almond), custard, flavoured milks, milkshakes, smoothies<br><br>Grains and flours (e.g. oats, rice, quinoa, wheat flour), breads, flat breads, sweet and savoury buns, pasta and noodles, breakfast cereals, porridge, savoury biscuits, scones, mixed dishes where cereal is the main ingredient, crumpets and pancakes, air popcorn<br><br>Fish and seafood (e.g. fresh, canned, mixed dishes), eggs, lean meats (e.g. beef, pork, lamb, kangaroo), poultry, organ meats, lean sausages, mixed meat or poultry dishes, meat alternatives (e.g. tofu), nuts and seeds<br><br>Margarine, table spreads, plant oils<br><br>Soups, homemade sauces, vinegar, yeast powder, intense sweeteners, herbs, spices, vanilla essence, gelatine	Population discretionary median intake = 0.0568<br><br>Population core median intake = 0.0867<br><br>1.526
Discretionary beverages			

Replace 50% of discretionary beverages (121.2g) with 104.5g of water or fruit and vegetable juices matching using replacement ratio of 0.862.	Fruit drinks, cordials, soft drinks (including sugar sweetened and artificially sweetened), flavoured mineral waters, sports drinks, energy drinks, sweetened water	Fruit and vegetable juice, water	Population discretionary median intake = 0.5881<br><br>Population core median intake = 0.5070<br><br>10.862
Replace 50% of discretionary beverages (121.2g) with 121.2g of water matching using replacement ratio of 1.000.	As above	Water	Population discretionary median intake =0.5881<br><br>Population core median intake = 42.3317<br><br>071.980 unfeasible ratio[4]: replaced by ratio of 1.000
Replace 50% of alcoholic beverages (130.4g) with 130.1g of water or fruit and vegetable juices matching using replacement ratio of 0.997.	Beer, wine, port, sherry, spirits, cider, mixed drinks and liqueur	Fruit and vegetable juice, water	Population discretionary median intake = 0.5083<br><br>Population core median intake = 0.5070<br><br>10.997
Replace 50% of "other" types of discretionary beverages (5.2g) with 5.5g of a healthier substitute (e.g. core variety tea/coffee) matching using replacement ratio of 1.051.	Chai latte, sweetened coffee, beverage base concentrate (e.g. chocolate, milo)	Black and herbal teas, coffee, reconstituted beverage base with water/milk, breakfast cereal beverage	Population discretionary median intake = 0.4238<br><br>Population core median intake = 0.4454<br><br>1.051

[1] Discretionary foods and beverages include only the items flagged as discretionary by the Australian Bureau of Statistics discretionary flag code [2] **[36]**

[2] Core choices include only items not flagged as discretionary by the Australian Bureau of Statistics discretionary flag code [2] **[36]**

[3] Replacement ratio was calculated by the population replacement median gram intake divided by the population discretionary median gram intake

[4] Due to the limited codes for plain water compared with other beverage codes and the aggregated population median intake of water, the replacement ratio of 71.980 was deemed unfeasible, therefore a replacement ratio of 1.00 was used which is consistent with the ratio of all discretionary beverages to all core beverages

Supplementary Table S2: Australian Health Survey population weighted mean base case intake nutrient density (amount of nutrient/1000 kJ) of adults aged 19 year and over

	**Total intake[1] original intake**	**Core choices original intake**	**Discretionary choices[2] original intake**	**Discretionary foods intake**	**Discretionary beverages intake**
Grams (g)	383.7	469.1	225.2	82.3	610.1
Energy (kJ)	1000.0	1000.0	1000.0	1000.0	1000.0
Protein (g)	10.5	13.4	5.1	6.2	1.0
Total Fat (g)	8.5	8.4	8.7	12.0	0.3
Saturated fat (g)	3.2	2.8	3.8	5.3	0.2
Carbohydrate (g)	26.0	25.7	26.4	25.9	28.0
Total Sugars (g)	11.8	9.1	16.9	14.1	24.4
Added sugars (g)	5.8	1.2	14.4	11.6	22.1
Sodium (mg)	279.4	277.2	283.7	366.6	73.2
Alcohol (g)	1.7	0.0	4.7	0.1	1807.0
Fiber (g)	2.6	3.5	1.0	1.3	0.1
Vitamin A retinol equivalents (µg)	97.9	129.5	39.3	49.4	9.4
Thiamine (vitamin B1) (mg)	0.2	0.2	0.1	0.1	0.0
Riboflavin (vitamin B2) (mg)	0.2	0.3	0.1	0.2	0.1
Niacin equivalents (mg)	4.8	5.9	2.6	2.9	1.7
Dietary folate equivalent (µg)	70.1	93.6	26.6	34.1	5.7
Vitamin B6 (pyridoxine) (mg)	0.2	0.2	0.1	0.1	0.3
Vitamin B12 (cobalamin) (µg)	0.5	0.7	0.2	0.3	0.1
Vitamin C (mg)	11.8	15.2	5.3	1.8	14.3
Vitamin E (mg)	1.2	1.4	0.9	1.2	0.1
Calcium (mg)	92.5	119.8	41.7	43.0	29.8
Iodine (µg)	19.8	25.9	8.5	8.3	7.7
Iron (mg)	1.3	1.6	0.7	0.8	0.4
Magnesium (mg)	38.9	48.5	21.2	18.7	25.4

Phosphorous (mg)	168.7	201.2	108.3	119.5	69.6
Potassium (mg)	334.8	414.8	186.3	190.1	168.0
Selenium (μg)	10.5	13.3	5.1	5.9	3.0
Zinc (mg)	1.3	1.6	0.6	0.7	0.1

[1] Total intake includes all core and discretionary foods and beverages

[2] Discretionary choices includes all discretionary foods and beverages

Supplementary Table S3: Modelled intakes simulating the impact on population mean nutrient profile of dietary strategies to reduce discretionary choices in Australian adults

	Moderation		Substitution				Reformulation	
	Reduction in discretionary choices[1]		Replacement of discretionary choices with core foods[2]		Replacement of discretionary choices with fruit and vegetables[3]		Reformulate discretionary choices[4]	
	Modelled intake[5]	% change in total intake[6]	Modelled intake	% change in total intake	Modelled intake	% change in total intake	Modelled intake	% change in total intake
Grams (g)	2991.5	-10.4	3367.9	0.9	3359.4	0.6	3323.0	-0.4
Energy (kJ)	7189.0	-17.3	7934.8	-8.8	7518.1	-13.6	8408.0	-3.3
Protein (g) (%E[7])	83.8 (19.5)	-7.9	93.7 (19.8)	3.0	86.1 (19.2)	-5.4	91.0 (18.1)	0.0
Total fat (g) (%E)	60.6 (31.7)	-17.9	66.8 (31.7)	-9.4	62.4 (31.3)	-15.4	73.8 (33.1)	0.0
Saturated fat (g) (%E)	21.9 (11.5)	-20.9	24.0 (11.4)	-13.4	22.3 (11.2)	-19.4	22.0 (9.9)	-20.6
Carbohydrate (g) (%E)	183.9 (43.3)	-17.7	205.3 (43.3)	-9.1	197.6 (44.0)	-12.5	225.9 (45.0)	0.0
Total sugars (g) (%E)	77.3 (18.0)	-24.9	84.0 (17.7)	-18.4	86.7 (19.3)	-15.7	91.9 (18.3)	-10.6
Added sugars (g) (%E)	28.7 (6.7)	-43.3	29.5 (6.2)	-41.7	29.0 (6.5)	-42.7	39.7 (7.9)	-21.7
Sodium (mg)	2001.3	-17.7	2210.6	-9.0	2050.4	-15.6	2270.4	-6.6
Alcohol (g) (%E)	7.2 (2.9)	-50.0	7.2 (2.7)	-50.0	7.2 (2.8)	-50.0	10.8 (3.8)	-25.0
Fiber (g)	21.4	-6.6	24.1	5.0	24.7	7.7	22.9	0.0
Vitamin A retinol equivalents (µg)	794.2	-6.8	891.3	4.6	964.4	13.2	851.8	0.0
Thiamine (vitamin B1) (mg)	1.3	-10.8	1.5	-0.4	1.4	-7.0	1.5	0.0
Riboflavin (vitamin B2) (mg)	1.7	-10.0	1.9	-0.6	1.8	-6.7	1.9	0.4
Niacin equivalents (mg)	37.6	-9.3	41.8	0.9	38.8	-6.4	41.4	0.0
Dietary folate equivalent (µg)	592.8	-5.9	624.3	5.0	629.9	6.9	609.9	0.0
Vitamin B6 (pyridoxine) (mg)	1.3	-12.7	1.5	-3.0	1.4	-5.4	1.5	0.0
Vitamin B12 (cobalamin) (µg)	4.1	-7.8	4.6	2.7	4.2	-7.2	4.5	-0.4
Vitamin C (mg)	94.3	-7.8	107.3	4.9	121.1	18.4	102.3	0.0
Vitamin E (mg)	9.2	-12.7	10.2	-2.8	9.9	-6.1	10.5	0.0
Calcium (mg)	745.3	-7.4	829.8	3.1	778.0	-3.3	804.6	0.0
Iodine (µg)	160.1	-7.1	178.6	3.6	163.5	-5.1	172.3	0.0
Iron (mg)	10.0	-9.5	11.2	1.1	10.7	-4.0	11.1	0.0
Magnesium (mg)	307.7	-9.2	342.6	1.2	330.2	-2.5	338.7	0.0

Phosphorous (mg)	1307.7	-10.9	1454.1	-0.9	1353.4	-7.7	1466.9	0.0
Potassium (mg)	2635.2	-9.5	2933.3	0.7	2920.6	0.3	2912.5	0.0
Selenium (μg)	83.3	-8.5	93.4	2.6	85.7	-5.8	91.0	0.0
Zinc (mg)	10.2	-7.7	11.4	3.4	10.6	-4.1	11.0	-0.4

[1] Reduction of discretionary choices by 50% with no energy compensation

[2] Replacement of 50% of discretionary foods with core foods and 50% of discretionary beverages with water, fruit and vegetable juices based on replacement ratio

[3] Replacement 50% of discretionary foods with fruit and vegetables and 50% of discretionary beverages with water, fruit and vegetable juices based on replacement ratio

[4] Reformulation by replacing 50% of saturated fat with unsaturated fat, reduce sugar by 25% and sodium by 20% in discretionary foods, and reduce sugar by 25% and alcohol by 25% in discretionary water-based and alcoholic beverages, and reduce sugar by 25% and sodium by 20% in "other" discretionary beverages

[5] Absolute values of modelled dietary intake of total core and discretionary food and beverages

[6] Percent change of total core and discretionary foods and beverages

[7] Modelled nutrient percentage of total energy intake

Supplementary Table S4: Sensitivity analyses testing the impact of manipulating discretionary choices intake on population mean nutrient profile

	Moderation				Reformulation			
	Moderate all discretionary choices (lower limit)[1]		Moderate all discretionary choices (upper limit)[2]		Reformulate as discretionary choices (lower limit)[3]		Reformulate discretionary choices (upper limit)[4]	
	Modelled dietary intake[5]	% change in total intake[6]	Modelled dietary intake	% change in total intake	Modelled dietary intake	% change in total intake	Modelled dietary intake	% change in total intake
Grams (g)	3034.2	-9.1	3119.5	-6.5	3329.3	-0.2	3303.1	-1.0
Energy (kJ)	7460.8	-14.2	8004.3	-8.0	8513.0	-2.1	8033.6	-7.6
Protein (g) (%E[7])	86.9 (19.5%)	-4.5	93.1 (19.5%)	2.3	91.0 (17.9%)	0.0	91.0 (19.0%)	0.0
Total fat (g) (%E)	63.2 (31.9%)	-14.4	68.3 (32.2%)	-7.4	73.8 (32.6%)	0.0	73.8 (34.6%)	0.0
Saturated fat (g) (%E)	22.9 (11.5%)	-17.4	24.8 (11.7%)	-10.5	24.8 (11.0%)	-10.3	19.1 (9.0%)	-30.9
Carbohydrate (g) (%E)	192.9 (43.3%)	-14.6	206.8 (43.2%)	-8.5	225.9 (44.4%)	0.0	225.9 (47.1%)	0.0
Total sugars (g) (%E)	79.9 (17.9%)	-22.3	85.3 (17.8%)	-17.1	98.2 (19.3%)	-4.6	76.1 (15.9%)	-26.0
Added sugars (g) (%E)	29.8 (6.7%)	-41.1	32.0 (6.7%)	-36.7	45.9 (9.0%)	-9.3	23.8 (5.0%)	-52.9
Sodium (mg)	2083.4	-14.3	2247.6	-7.5	2350.1	-3.3	2110.4	-13.2
Alcohol (g) (%E)	7.2 (2.8%)	-50.0	7.2 (2.6%)	-50.0	10.8 (3.7%)	-25.0	7.2 (2.6%)	-49.9
Fiber (g)	22.2	-3.1	23.8	3.7	22.9	0.0	22.9	0.0
Vitamin A retinol equivalents (μg)	822.8	-3.4	880.2	3.3	851.8	0.0	851.8	0.0
Thiamine (vitamin B1) (mg)	1.4	-7.4	1.5	-0.6	1.5	0.0	1.5	0.0
Riboflavin (vitamin B2) (mg)	1.8	-7.0	1.9	-0.9	1.9	0.3	1.9	0.4
Niacin equivalents (mg)	38.9	-6.1	41.6	0.4	41.4	0.0	41.4	0.0
Dietary folate equivalent (μg)	601.2	-3.0	618.1	2.8	609.9	0.0	609.9	0.0
Vitamin B6 (pyridoxine) (mg)	1.4	-9.8	1.4	-4.0	1.5	0.0	1.5	0.0
Vitamin B12 (cobalamin) (μg)	4.3	-4.6	4.6	1.9	4.5	-0.3	4.5	-0.4
Vitamin C (mg)	96.7	-5.5	101.5	-0.8	102.3	0.0	102.3	0.0
Vitamin E (mg)	9.5	-9.2	10.3	-2.3	10.5	0.0	10.5	0.0
Calcium (mg)	769.6	-4.4	818.1	1.7	804.6	0.0	804.6	0.0
Iodine (μg)	165.2	-4.1	175.4	1.8	172.3	0.0	172.3	0.0
Iron (mg)	10.4	-6.2	11.2	0.5	11.1	0.0	11.1	0.0
Magnesium (mg)	317.1	-6.4	335.9	-0.8	338.7	0.0	338.7	0.0
Phosphorous (mg)	1355.4	-7.6	1450.7	-1.1	1466.9	0.0	1466.9	0.0

Potassium (mg)	2724.6	-6.5	2903.4	-0.3	2912.5	0.0	2912.5	0.0
Selenium (µg)	86.2	-5.3	92.0	1.1	91.0	0.0	91.0	0.0
Zinc (mg)	10.5	-4.3	11.3	2.6	11.0	-0.3	11.0	-0.4

[1] Decrease all discretionary foods by 50% plus include energy compensation of 25% of all foods (core and discretionary foods) and decrease all discretionary beverages by 50% with no compensation

[2] Decrease all discretionary foods by 50% plus include energy compensation of 75% of all foods (core and discretionary foods) and decrease all discretionary beverages by 50% with no compensation

[3] Reformulate discretionary foods by replacing 25% saturated fat for unsaturated fat, reducing added sugar by 10% and sodium by 10%; and reformulate discretionary beverages by reducing added sugar by 10% in water-based beverages, reducing sugar by 25% and sodium by 20% in other discretionary beverages, and reducing sugar by 25% alcohol content by 25% in alcoholic beverages

[4] Reformulate discretionary foods by replacing 75% saturated fat for unsaturated fat, reducing added sugar by 40% and sodium by 40%; and reformulate discretionary beverages by replacing 100% added sugar with 100% with artificial sweetener in water-based beverages, reducing sugar by 100% (50% replaced with artificial sweetener) and sodium by 40% in other discretionary beverages, and reducing alcohol content by 50% in alcoholic beverages

[5] Absolute values of modelled dietary intake of total core and discretionary food and beverages

[6] Percent change of total core and discretionary foods and beverages

[7] Modelled nutrient percentage of total energy intake

Supplementary Table S5: Sensitivity analyses testing the impact of moderating discretionary foods on population mean nutrient profile

	Moderation of all discretionary foods (lower limit)[1]		Moderation of all discretionary foods (upper limit)[2]	
	Modelled dietary profile	% change in total intake[3]	Modelled dietary profile	% change in total intake
Grams (g)	3291.0	-1.4	3376.3	1.2
Energy (kJ)	7882.6	-9.4	8426.1	-3.1
Protein (g) (%E[4])	87.3 (18.5%)	-4.0	93.5 (18.6%)	2.8
Total fat (g) (%E)	63.3 (30.3%)	-14.2	68.5 (30.6%)	-7.2
Saturated fat (g) (%E)	22.9 (11.0%)	-17.2	24.9 (11.1%)	-10.2
Carbohydrate (g) (%E)	204.7 (43.5%)	-9.4	218.6 (43.4%)	-3.2
Total sugars (g) (%E)	90.2 (19.2%)	-12.3	95.6 (19.0%)	-7.1
Added sugars (g) (%E)	39.1 (8.3%)	-22.7	41.3 (8.2%)	-18.3
Sodium (mg)	2114.2	-13.0	2278.4	-6.3
Alcohol (g) (%E)	14.4 (5.3%)	0.0	14.4 (5.0%)	0.0
Fiber (g)	22.3	-2.8	23.8	4.0
Vitamin A retinol equivalents (µg)	826.8	-2.9	884.2	3.8
Thiamine (vitamin B1) (mg)	1.4	-6.8	1.5	0.0
Riboflavin (vitamin B2) (mg)	1.8	-5.7	1.9	0.4
Niacin equivalents (mg)	39.6	-4.3	42.3	2.1
Dietary folate equivalent (µg)	603.6	-2.2	620.4	3.7
Vitamin B6 (pyridoxine) (mg)	1.5	-2.6	1.5	3.3
Vitamin B12 (cobalamin) (µg)	4.3	-3.3	4.6	3.2
Vitamin C (mg)	102.7	0.4	107.6	5.1
Vitamin E (mg)	9.6	-8.9	10.3	-2.0
Calcium (mg)	782.1	-2.8	830.7	3.2
Iodine (µg)	168.4	-2.2	178.7	3.7
Iron (mg)	10.6	-4.5	11.3	2.2
Magnesium (mg)	327.8	-3.2	346.6	2.3
Phosphorous (mg)	1384.6	-5.6	1479.9	0.9

Potassium (mg)	2795.3	-4.0	2974.1	2.1
Selenium (µg)	87.5	-3.9	93.3	2.5
Zinc (mg)	10.6	-3.7	11.3	3.2

[1] Reduction of all discretionary foods by 50% plus include energy compensation of 25% of all foods (core and discretionary foods)

[2] Reduction of all discretionary foods by 50% plus include energy compensation of 75% of all foods (core and discretionary foods)

[3] Percent change of total core and discretionary foods and beverages

[4] Modelled nutrient percentage of total energy intake

Note: No compensation was assumed for discretionary beverages, therefore discretionary beverage results can be found in manuscript Table 2.

Supplementary Table S6: Sensitivity analyses testing the impact of substituting water-based discretionary beverages to core beverages on population mean nutrient profile

	Replace 50% of water-based discretionary beverages with water, or fruit and vegetable juices		Replace 50% of water-based discretionary beverages with water	
	Modelled dietary profile	Percent change in total intake[1]	Modelled dietary profile	Percent change in total intake
Grams (g)	3320.9	-0.5	3337.7	0.0
Energy (kJ)	8557.5	-1.6	8551.3	-1.7
Protein (g) (%E[2])	91.0 (17.8)	0.0	91.0 (17.8)	0.0
Total fat (g) (%E)	73.8 (32.5)	0.0	73.8 (32.5)	0.0
Saturated fat (g) (%E)	27.7 (12.2)	0.0	27.7 (12.2)	0.0
Carbohydrate (g) (%E)	217.5 (42.5)	-3.7	217.2 (42.5)	-3.9
Total sugars (g) (%E)	94.5 (18.5)	-8.1	94.2 (18.4)	-8.5
Added sugars (g) (%E)	42.3 (8.3)	-16.4	42.3 (8.3)	-16.4
Sodium (mg)	2417.3	-0.5	2417.3	-0.5
Alcohol (g) (%E)	14.4 (4.9)	0.0	14.4 (4.9)	0.0
Fiber (g)	22.9	0.1	22.9	-0.1
Vitamin A retinol equivalents (µg)	851.8	0.0	850.2	-0.2
Thiamine (vitamin B1) (mg)	1.5	0.1	1.5	0.0
Riboflavin (vitamin B2) (mg)	1.9	-0.8	1.9	-0.9
Niacin equivalents (mg)	41.1	-0.6	41.1	-0.7
Dietary folate equivalent (µg)	287.6	0.0	286.1	-0.6
Vitamin B6 (pyridoxine) (mg)	1.4	-3.9	1.4	-4.0
Vitamin B12 (cobalamin) (µg)	4.5	-1.0	4.5	-1.0
Vitamin C (mg)	100.5	-1.7	98.6	-3.6
Vitamin E (mg)	10.5	-0.2	10.5	-0.2
Calcium (mg)	803.5	-0.1	803.4	-0.2
Iodine (µg)	170.7	-1.0	170.7	-0.9
Iron (mg)	11.1	-0.2	11.1	-0.2

Magnesium (mg)	338.9	0.1	338.9	0.1
Phosphorous (mg)	1457.5	-0.6	1456.9	-0.7
Potassium (mg)	2903.9	-0.3	2897.2	-0.5
Selenium (μg)	90.8	-0.2	90.9	-0.2
Zinc (mg)	11.0	-0.1	11.0	-0.1

[1] Percent change of total core and discretionary foods and beverages

[2] Modelled nutrient percentage of total energy intake

Supplementary Table S7: Sensitivity analyses testing the impact of reformulating discretionary foods or beverages on population mean nutrient profile

	**Reformulate discretionary foods (lower limit)**[1]		**Reformulate discretionary foods (upper limit)**[2]		**Reformulate discretionary beverages (lower limit)**[3]		**Reformulate discretionary beverages (upper limit)**[4]	
	**Modelled dietary profile**	**% change in total intake**[5]	**Modelled dietary profile**	**% change in total intake**	**Modelled dietary profile**	**% change in total intake**	**Modelled dietary profile**	**% change in total intake**
Grams (g)	3335.1	-0.1	3327.3	-0.3	3331.9	-0.1	3313.5	-0.7
Energy (kJ)	8656.6	-0.5	8528.8	-1.9	8555.5	-1.6	8202.9	-5.7
Protein (g) (%E[6])	91.0 (17.6%)	0.0	91.0 (17.9%)	0.0	91.0 (17.8%)	0.0	91.0 (18.6%)	0.0
Total fat (g) (%E)	73.8 (32.1%)	0.0	73.8 (32.6%)	0.0	73.8 (32.5%)	0.0	73.8 (33.9%)	0.0
Saturated fat (g) (%E)	24.9 (10.8%)	-10.3	19.1 (8.4%)	-30.9	27.7 (12.2%)	0.0	27.7 (12.7%)	0.0
Carbohydrate (g) (%E)	225.9 (43.7%)	0.0	225.9 (44.3%)	0.0	225.9 (44.2%)	0.0	225.9 (46.1%)	0.0
Total sugars (g) (%E)	100.3 (19.4%)	-2.5	92.8 (18.2%)	-9.8	100.7 (19.7%)	-2.1	86.2 (17.6%)	-16.2
Added sugars (g) (%E)	48.1 (9.3%)	-5.0	40.5 (7.9%)	-19.9	48.4 (9.5%)	-4.3	33.9 (6.9%)	-33.0
Sodium (mg)	2350.8	-3.3	2111.8	-13.1	2429.8	0.0	2429.1	-0.1
Alcohol (g) (%E)	14.4 (4.9%)	0.0	14.4 (4.9%)	0.0	10.8 (3.7%)	-25.0	7.2 (2.6%)	-49.9
Fiber (g)	22.9	0.0	22.9	0.0	22.9	0.0	22.9	0.0
Vitamin A retinol equivalents (µg)	851.8	0.0	851.8	0.0	851.8	0.0	851.8	0.0
Thiamine (vitamin B1) (mg)	1.54	0.0	1.54	0.0	1.5	0.0	1.5	0.0
Riboflavin (vitamin B2) (mg)	1.89	0.1	1.89	0.1	1.9	0.2	1.9	0.3
Niacin equivalents (mg)	41.4	0.0	41.4	0.0	41.4	0.0	41.4	0.0
Dietary folate equivalent (µg)	609.9	0.0	609.9	0.0	609.9	0.0	609.9	0.0
Vitamin B6 (pyridoxine) (mg)	1.50	0.0	1.50	0.0	1.5	0.0	1.5	0.0
Vitamin B12 (cobalamin) (µg)	4.54	-0.1	4.54	-0.1	4.5	-0.2	4.5	-0.3
Vitamin C (mg)	102.3	0.0	102.3	0.0	102.3	0.0	102.3	0.0
Vitamin E (mg)	10.5	0.0	10.5	0.0	10.5	0.0	10.5	0.0
Calcium (mg)	804.6	0.0	804.6	0.0	804.6	0.0	804.6	0.0
Iodine (µg)	172.3	0.0	172.3	0.0	172.3	0.0	172.3	0.0
Iron (mg)	11.1	0.0	11.1	0.0	11.1	0.0	11.1	0.0
Magnesium (mg)	338.7	0.0	338.7	0.0	338.7	0.0	338.7	0.0
Phosphorous (mg)	1466.9	0.0	1466.6	0.0	1466.9	0.0	1466.9	0.0
Potassium (mg)	2912.5	0.0	2912.5	0.0	2912.5	0.0	2912.5	0.0

Selenium (μg)	91.0	0.0	91.0	0.0	91.0	0.0	91.0	0.0
Zinc (mg)	10.98	-0.1	10.98	-0.1	11.0	-0.2	11.0	-0.3

[1] Reformulate discretionary foods by replacing 25% saturated fat with equivalent gram of unsaturated fat, reducing added sugar by 10% and reducing sodium by 10%

[2] Reformulate discretionary foods by replacing 75% saturated fat with equivalent gram of unsaturated fat, reducing added sugar by 40% and reducing sodium by 40%

[3] Reformulate discretionary beverages by reducing added sugar by 10% in water-based beverages, reducing sugar by 25% and sodium by 20% in other discretionary beverages, and reducing sugar by 25% alcohol content by 25% in alcoholic beverages

[4] Reformulate discretionary beverages by replacing 100% added sugar with 100% with artificial sweetener in water-based beverages, reducing sugar by 100% (50% replaced with artificial sweetener) and sodium by 40% in other discretionary beverages, and reducing alcohol content by 50% in alcoholic beverages

[5] Percent change of total core and discretionary foods and beverages

[6] Modelled nutrient percentage of total energy intake
